# Case report: Almonertinib in combination with bevacizumab for leptomeningeal metastases from epidermal growth factor receptor-mutation non-small cell lung cancer: Case series

**DOI:** 10.3389/fonc.2022.1040450

**Published:** 2022-11-10

**Authors:** Yuhai Zhang, Meilin Zhang, Wanwan Cheng, Shencun Fang

**Affiliations:** ^1^ Department of Neurosurgery, The Affiliated Brain Hospital of Nanjing Medical University, Nanjing, China; ^2^ Department of Oncology, The First Affiliated Hospital of Nanjing Medical University, Nanjing, China; ^3^ Department of Respiratory Medicine, Nanjing Chest Hospital, The Affiliated Brain Hospital of Nanjing Medical University, Nanjing, China

**Keywords:** leptomeningeal metastasis, non-small cell lung cancer, almonertinib, bevacizumab, EGFRm

## Abstract

Leptomeningeal metastasis (LM) is a lethal complication of advanced non-small cell lung cancer (NSCLC) with rapid deterioration and poor prognosis. It has no standard treatment for epidermal growth factor receptor mutation (EGFRm) NSCLC, and improving the clinical outcomes for patients with LM has become an urgent problem in clinical treatment. Both almonertinib and bevacizumab are capable of crossing the blood–brain barrier with comparable central nervous system effectiveness. To date, the almonertinib treatment in combination with bevacizumab in EGFRm NSCLC with LM has not been studied. We herein present five cases to further evaluate the effectiveness and tolerability of almonertinib in combination with bevacizumab for patients with EGFRm NSCLC and LM. For the first time, we report that almonertinib plus bevacizumab can not only effectively improve the neurological symptoms caused by LM but also prolong the survival time of patients with limited and controllable side effects, which provided a novel therapeutic approach for LM from EGFRm NSCLC.

## Introduction

Leptomeningeal metastasis (LM) is a lethal complication of advanced non-small cell lung cancer (NSCLC) in which cancer metastasizes to the leptomeninges, subarachnoid space, and other cerebrospinal fluid (CSF) compartments (1). LM incidence is increasing, reaching 3%–5% of patients with advanced NSCLC, and occurring in up to 9.4% of patients with epidermal growth factor receptor mutation (EGFRm) (2–4). Patients with LM from NSCLC have rapid deterioration and poor prognosis with a median overall survival (OS) of 3–10 months (2). LM with EGFRm NSCLC has no standard treatment, although whole-brain radiotherapy, intrathecal chemotherapy, ventriculoperitoneal shunt, and molecularly targeted therapies were described (1). Recently, EGFR tyrosine kinase inhibitors (TKIs) are a potential therapeutic option for patients with EGFRm NSCLC and LM. Preclinical evidence shows that third-generation EGFR-TKIs, investigated as an LM therapy, have greater blood–brain barrier (BBB) penetration than first- and second-generation treatment (5, 6). Multiple clinical studies have revealed that third-generation EGFR-TKIs demonstrated a promising therapeutic efficacy in improving imaging response and neurological function in patients with LM from EGFRm advanced NSCLC (7–9).

Bevacizumab is a monoclonal humanized antibody against vascular endothelial growth factor (VEGF) and inhibits tumor angiogenesis by blocking VEGF. The biologically synergistic antitumor activity of dual blockade of both EGFR and VEGF pathways in EGFRm advanced NSCLC has been demonstrated in preclinical studies (10, 11). In addition, several clinical trials have confirmed that EGFR-TKIs combined with bevacizumab significantly improve progression-free survival (PFS) than EGFR-TKIs monotherapy for patients with EGFRm NSCLC, especially in those with central nervous system (CNS) metastasis (11, 12).

Recently, almonertinib, a novel third-generation EGFR-TKI, was approved in China as a first-line treatment for advanced NSCLC with EGFR ex19del or L858R mutations and as a second-line treatment for EGFR T790M-positive NSCLC. Preclinical studies showed that almonertinib easily penetrates the BBB and inhibits brain and spinal cord metastasis in advanced NSCLC (6). To date, almonertinib treatment combined with bevacizumab in EGFRm NSCLC with LM has not been studied. We herein presented five cases to further evaluate the effectiveness and tolerability of almonertinib with bevacizumab for patients with EGFRm NSCLC and LM.

## Presentation

### Case 1

**Figure 1:**
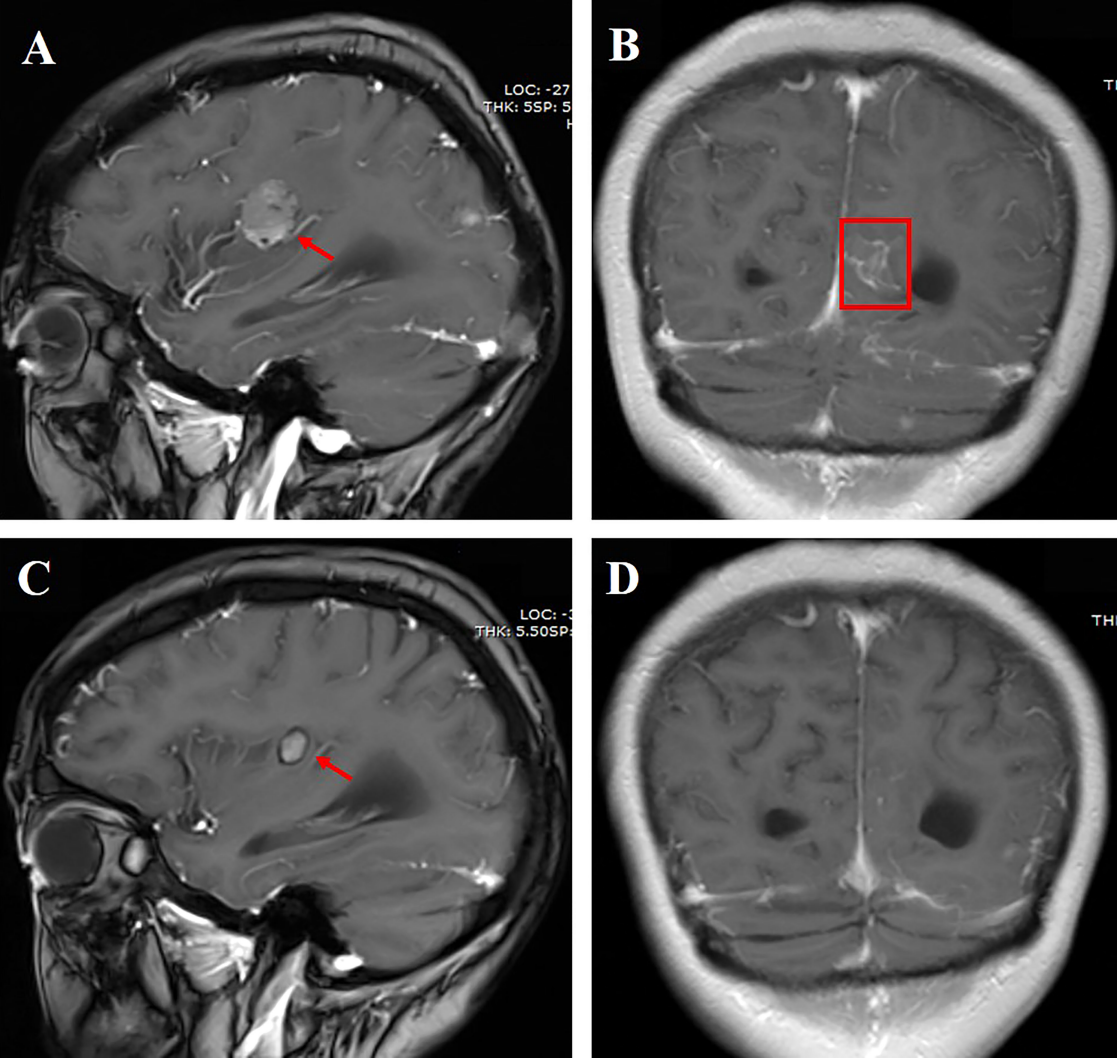
**(A)** Cranial enhanced MRI showing the right parietal lobe mass measuring approximately 2.1 cm (red arrow); **(B)** linear enhancement of leptomeninges in the left cerebral hemisphere (red box); **(C)** the right parietal lobe mass shrank 6 months after aumolertinib plus bevacizumab treatment; **(D)** linear enhancement of leptomeninges disappeared 6 months after aumolertinib plus bevacizumab treatment.

A 76-year-old female nonsmoker was diagnosed with stage IV lung adenocarcinoma, with primary tumors of 4.7 cm in the left upper lobe. **Table 1** shows the demographic profiles. LM and multiple metastases in the bilateral cerebral hemisphere, cerebellar hemisphere, and brainstem were diagnosed based on cranial enhanced magnetic resonance imaging (MRI). LM was diagnosed by CSF cytology. The subsequent lung tissue sample genotype revealed EGFR L858R mutation by amplification refractory mutation system. She suffered dizziness, headache, nausea, and vomiting during hospitalization. Almonertinib (110 mg/day) plus bevacizumab (7.5 mg/kg, 21-day cycle) was then prescribed as the first-line treatment. Surprisingly, her LM-related symptoms significantly improved, and the Eastern Cooperative Oncology Group performance status (PS) score improved from 4 to 2. Moreover, an obvious diminution was observed both in primary lesions and brain metastases after 6 weeks of treatment. Following 3 months of treatment, leptomeningeal metastases showed a marked shrinkage, with complete disappearance after 6 months according to the modified Response Assessment in Neuro-Oncology-LM radiological criteria (13) (**Figure 1**). Furthermore, the effect of the combination therapy has lasted for >22 months and is still in close follow-up. The regimen was well tolerated, with mild hypertension and prolongation of activated partial thromboplastin time (APTT) during follow-up.

**Table 1:** Patient characteristics.

Patient	Age	Gender	Histology	Brain metastasis	Extracranial lesions	EGFR mutation	Concomitant mutations	Neurological symptoms
1	76	Female	Adenocarcinoma	Multiple, LM	Lung, bone	EGFR L858R		Dizziness, headache nausea and vomiting
2	50	Female	Adenocarcinoma	Multiple, LM	Lung, liver, bone	EGFR 19del	CDKN2A, TP53, PTEN	Dizziness, headache gait disorder, vomiting
3	54	Female	Adenocarcinoma	LM		EGFR 19del	CDK4	Dizziness, headache
4	59	Male	Adenocarcinoma	Multiple, LM	Lung, bone	EGFR 19del		Dizziness, headache, cognitive impairment
5	52	Female	Adenocarcinoma	LM	Lung, bone	EGFR T790M		Dizziness, headache

### Case 2

**Figure 2:**
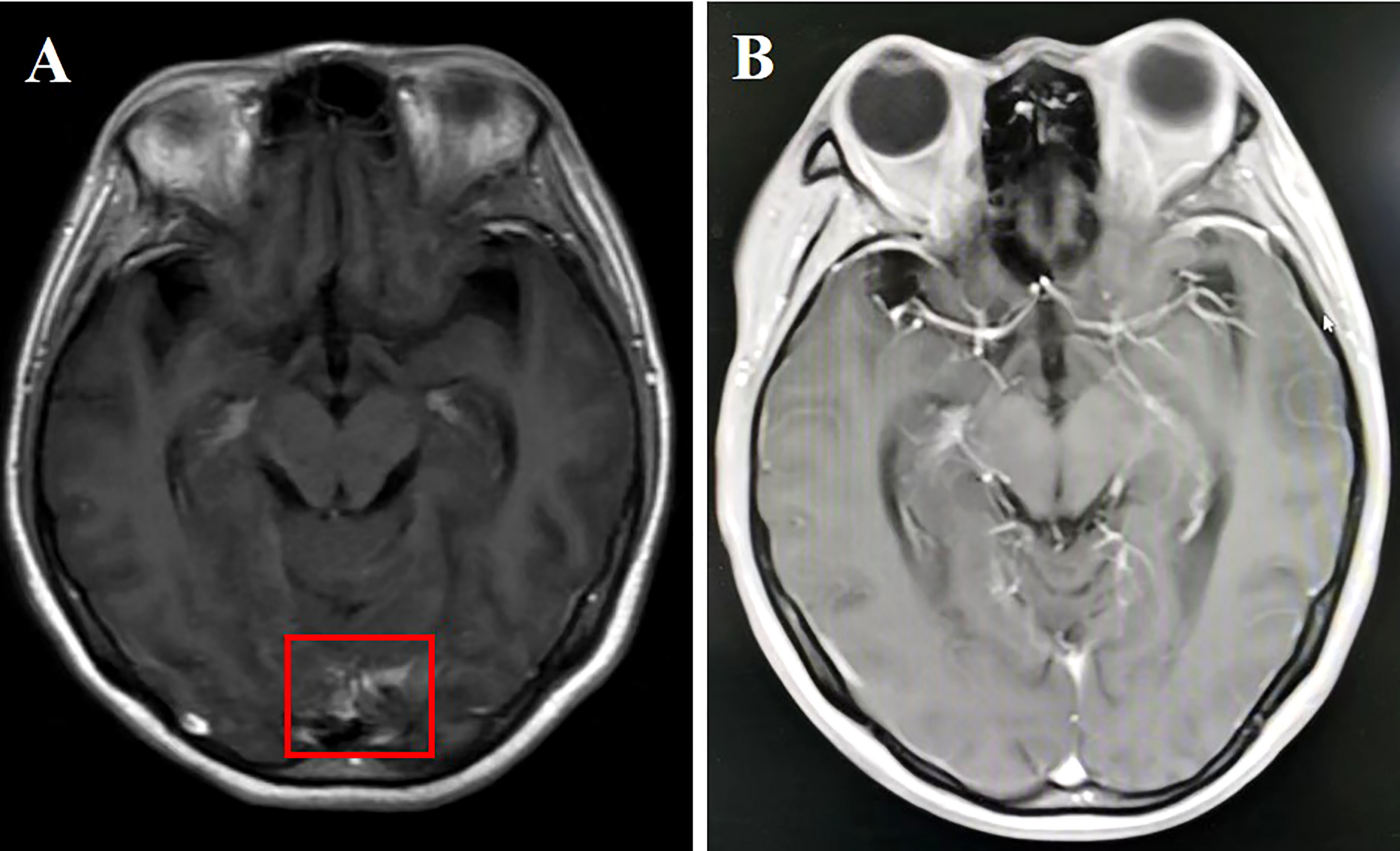
**(A)** Cranial enhanced MRI showing linear enhancement of leptomeninges in the temporal lobe (red box); **(B)** linear enhancement of leptomeninges disappeared 2 months after aumolertinib plus bevacizumab treatment.

A 50-year-old female nonsmoker was diagnosed with right lower lobe lung adenocarcinoma (cT4N2M1b) in December 2020. Positron emission tomography-computed tomography (CT) scanning revealed extensive systemic metastases, including multiple bones, liver, and brain metastases. A contrast-enhanced brain MRI demonstrated multiple enhancing hyperintense lesions surrounded by edema in her brain, with associated meningeal enhancement. CSF cytology showed the presence of malignant cells. DNA sequencing of her CSF revealed an EGFR mutation (exon 19 deletion), with CDKN2A, TP53, and PTEN comutation. Almonertinib (110 mg/day) plus bevacizumab (7.5 mg/kg, 21-day cycle) was implemented for 2 months, which improved LM-related symptoms and cleared CSF cytology. Moreover, the PS of the patient improved from 4 to 1. A repeated MRI revealed significant parenchymal and leptomeningeal lesion improvements (**Figure 2**). Response in the CNS has been maintained for >11 months after the combination therapy initiation. However, this patient eventually succumbed to a cerebral hernia caused by rapid LM progression in December 2021.

### Case 3

**Figure 3:**
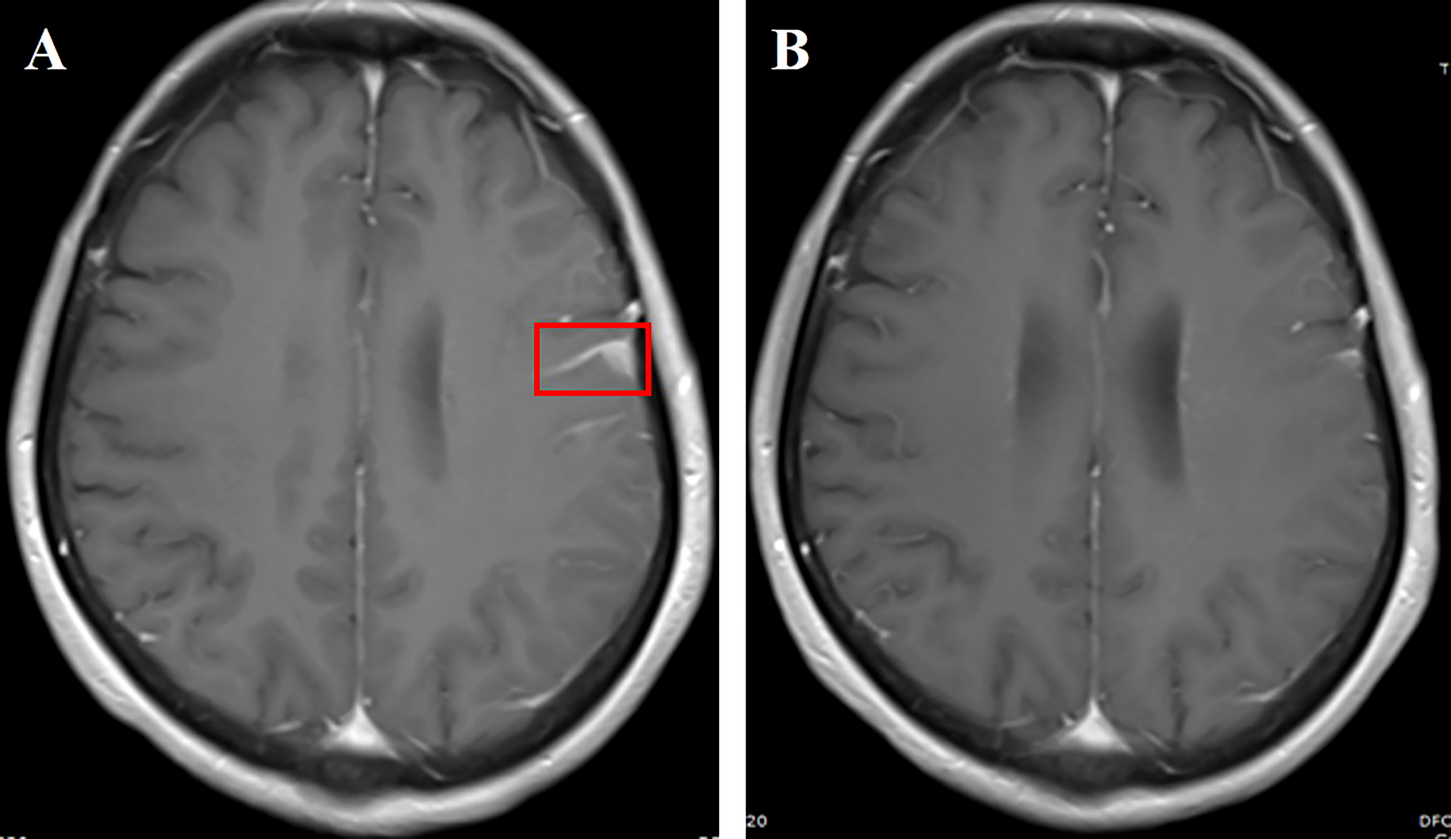
**(A)** Cranial enhanced MRI showing left frontotemporal linear enhancement (red box); **(B)** linear enhancement of leptomeninges shrunk 2 months after aumolertinib plus bevacizumab treatment.

A 54-year-old female nonsmoker underwent left lung cancer surgical resection, with a diagnosis of stage IIIA (pT1N2M0) lung adenocarcinoma. She received four cycles of pemetrexed plus cisplatin as adjuvant chemotherapy. Two years later, she attended the hospital with dizziness, headache, and vomiting in June 2021, and enhanced leptomeningeal dissemination was found by brain MRI. However, no other metastatic lesions were revealed by combined results obtained from thoracic and abdominal CT and whole-body bone scan. LM was confirmed based on brain MRI and positive CSF cytology. DNA sequencing of the CSF specimen revealed an EGFR mutation (exon 19 deletion) with CDK4 amplification. Almonertinib (110 mg/day) plus bevacizumab (7.5 mg/kg, 21-day cycle) was administered for 1 month, which resulted in neurological symptom disappearance and PS improvement. A remarkable LM shrinkage was observed after 2 months of combination treatment (**Figure 3**). Furthermore, the efficacy has been durable for 10 months without any notable side effects. Re-examination of the cranial MRI due to headache recurrence in May 2022 showed meningeal metastasis progression.

### Case 4

**Figure 4:**
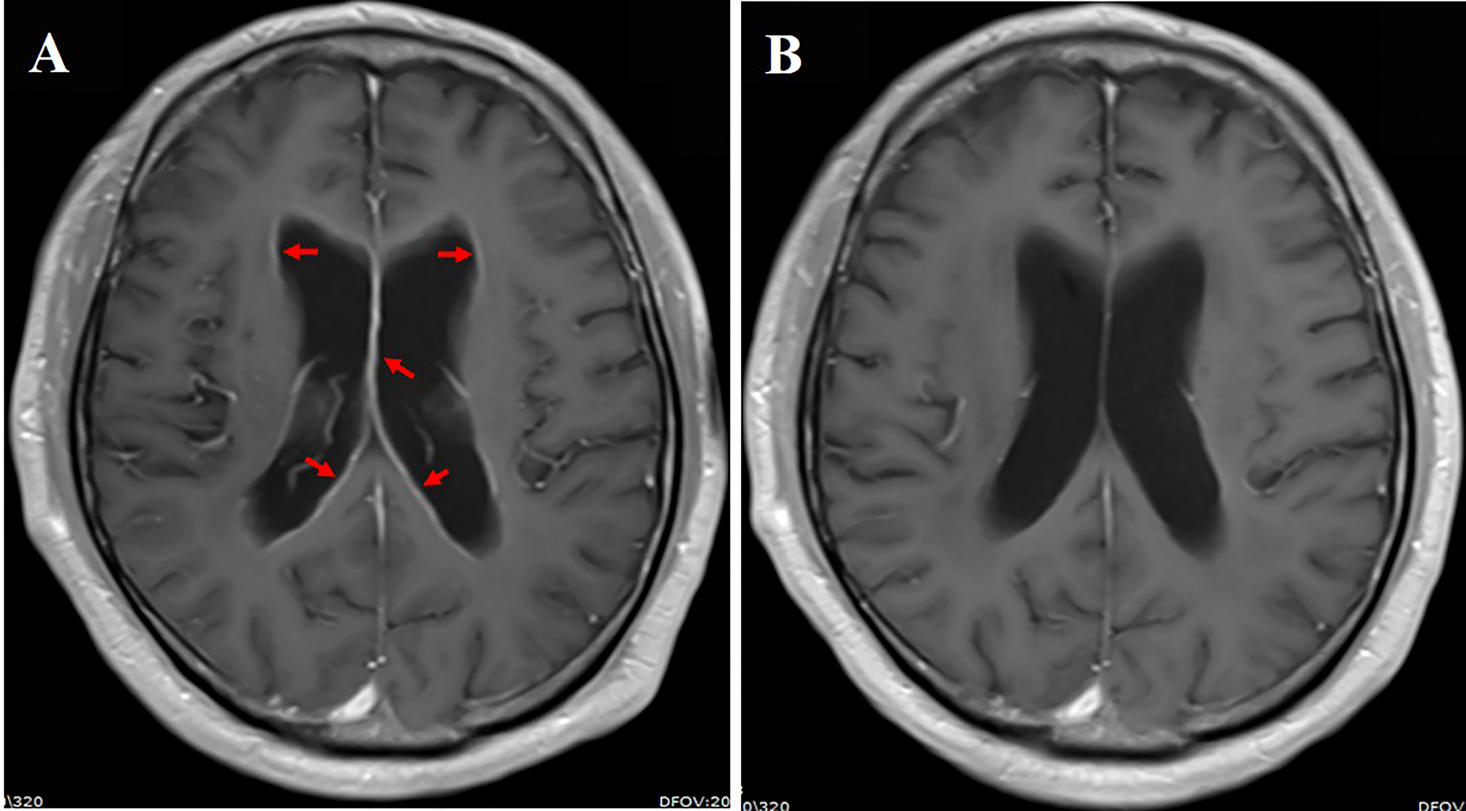
**(A)** Cranial enhanced MRI showing linear enhancement of ventricular ependyma (red arrow); **(B)** linear enhancement of leptomeninges regressed 2 months after aumolertinib plus bevacizumab treatment.

A 59-year-old male smoker was diagnosed with metastatic NSCLC adenocarcinoma that involved multiple lungs, bone, and symptomatic brain metastases in February 2021. Multiple brain metastases and abnormal signals on leptomeninges were demonstrated in cranial enhanced MRI. He developed a headache and cognitive impairmentand was diagnosed with LM based on the clinical presentation, imaging findings, and CSF cytological analysis. Primary tumor specimen DNA sequencing revealed an EGFR mutation (exon 19 deletion). He was then treated with almonertinib (110 mg/day) plus bevacizumab (7.5 mg/kg, 21-day cycle) as the first-line treatment. LM-related symptoms significantly diminished within a month and the PS improved from 4 to 2. Leptomeningeal dissemination and brain metastases had regressed on MRI 2 months after the treatment initiation (**Figure 4**). The lung and bone metastases continued to synchronously decrease in size. In addition, this partial remission has been sustained for >15 month and remained in close follow-up. Elevated blood creatine phosphokinase (CPK) and transaminase levels were reported. However, the side effects were mild and did not require any treatment.

### Case 5

**Figure 5:**
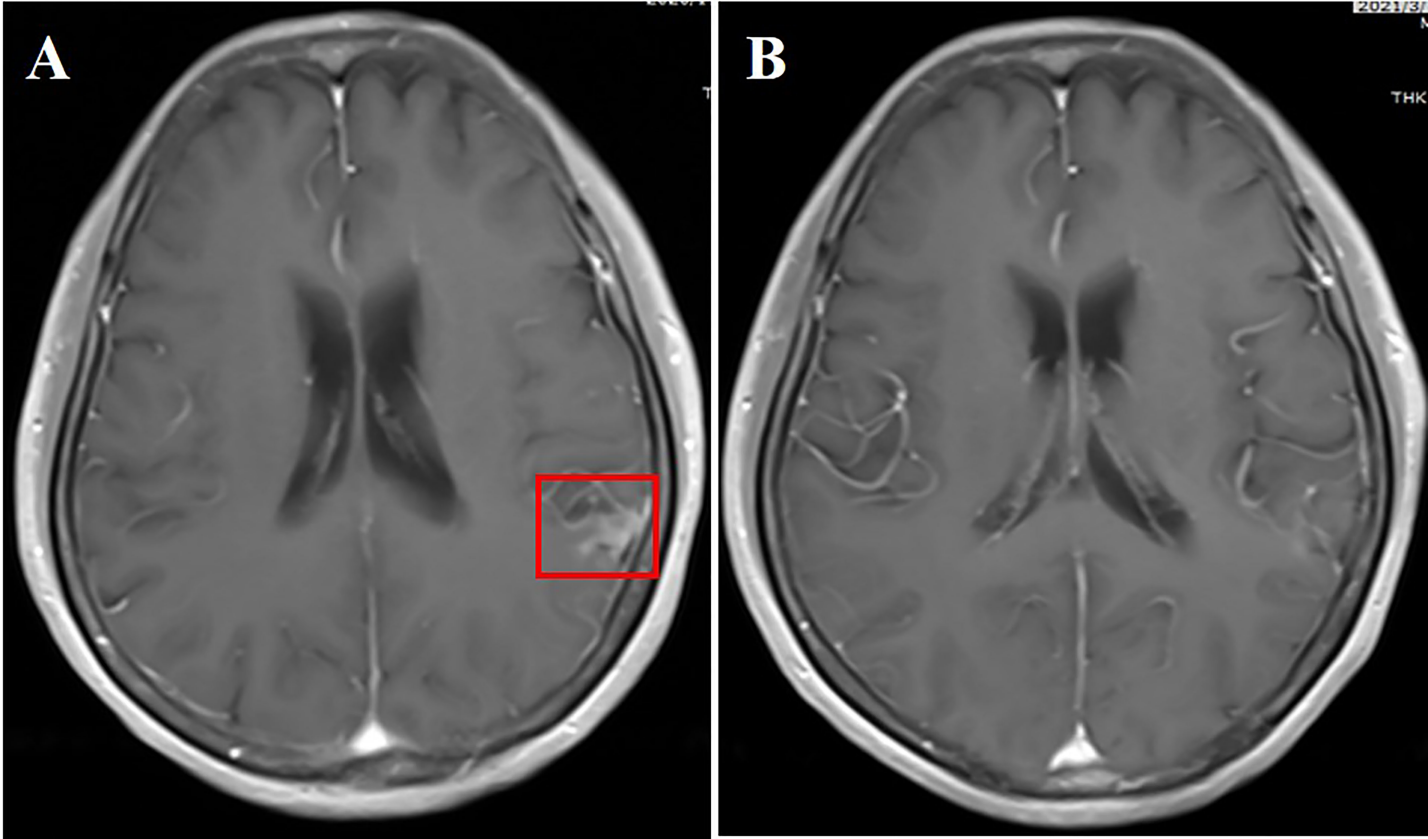
**(A)** Cranial enhanced MRI showing patchy high signals in the left temporal lobe (red box); **(B)** high signals of leptomeninges disappeared 2 months after aumolertinib plus bevacizumab treatment.

A 52-year-old female nonsmoker underwent left lung cancer surgical resection in February 2015, with a diagnosis of stage IIB (pT2N1M0) lung adenocarcinoma harboring an EGFR del-19 mutation. She received four cycles of adjuvant chemotherapy with pemetrexed plus cisplatin. Treatment with gefitinib at 250 mg daily was started for mediastinal lymph node and left lung metastasis in February 2016. The efficacy was evaluated as complete response (CR) after two cycles of gefitinib therapy. In October 2020, she started to experience dizziness and headache. Cranial enhanced MRI indicated high signal intensities on the left occipital lobe and adjacent meninges. CSF cytology found adenocarcinoma cells. DNA sequencing of the CSF specimen revealed an EGFR T790M mutation, and her PS score was 2. She was then treated with almonertinib (110 mg/day) plus bevacizumab (7.5 mg/kg, 21-day cycle) as the second-line treatment. LM-related symptoms disappeared within a month and the PS improved from 2 to 0. Leptomeningeal metastases showed a complete disappearance after 2 months of treatment (**Figure 5**). The therapeutic effect has lasted for >18 months and is still in close follow-up. She complained of grade 1 diarrhea without other adverse events.

## Discussion

This study used almonertinib combined with bevacizumab as the first-line treatment in four patients with LM with EGFR mutations and as a second-line treatment in one patient with LM with T790M-positive. The findings showed that almonertinib plus bevacizumab, whether as first- or second-line therapy, can not only effectively improve the LM-related neurological symptoms but also prolong the survival time of patients (**Table 2**). Although LM in two patients (NO. 2 and NO. 3) recurred, the three patients remained on maintenance therapy. Our case series illustrated the value of almonertinib combined with bevacizumab as treatment in patients with LM in EGFR mutation.

**Table 2:** Tumor responses and disease control.

Patient	Change of PS	Best systemic response	Best radiographic LM response	Neurological symptomatic response	PFS (months)	adverse events
1	4→2	PR	Disappeared	Improved	Stable (22 FU)	Hypertension and prolonged APTT
2	4→1	PR	Improved	Improved	11 (died)	Rash
3	3→0	NA	Improved	Disappeared	10 (alive)	
4	4→2	PR	Improved	Improved	Stable (15 FU)	Elevated CPK and transaminase
5	2→0	NA	Disappeared	Disappeared	Stable (18 FU)	Diarrhea

NA, not available; SD, stable disease; PR, partial response; CR, complete response; FU, follow-up.

The circulating cell-free DNA (cfDNA) in the CSF cannot fully circulate in the blood system due to the blood–CSF barrier existence, resulting in an extremely small amount of cfDNA from the CNS being released into the plasma. Therefore, the gene expression profiles in plasma could not fully represent the “real world” of LM (14). CSF, as a liquid biopsy medium, has been successfully used in patients with NSCLC with LM. Many studies have demonstrated that more comprehensive profiles of driver and resistance genes were shown in the CSF than in plasma and primary lesions at LM diagnosis (15, 16). CSF genotyping has a unique value in identifying the heterogeneous therapeutic effects of EGFR-TKIs on patients with NSCLC with LM. For example, concurrent cell cycle pathway alterations (CDK4 and CDKN2A) in the CSF were the poor prognosis indicators for osimertinib treatment, but not in the plasma (17). The relatively short PFS of patients NO. 2 and NO. 3 in our study may be due to the comutations of CDKN2A and CDK4 in the CSF, respectively, which further supported the above conclusions.

Osimertinib effectively penetrates the BBB and therefore represents a promising therapeutic option for patients with EGFRm NSCLC with LM. The BLOOM study reported that the median PFS of a double osimertinib dosage in patients with EGFRm with LM was 8.6 months and the median OS was 11.0 months (9). Osimertinib monotherapy leads to tumor shrinkage and improves prognosis, but disease progression is inevitable. Combined treatment strategies may either delay or prevent resistance and progression. Both osimertinib and bevacizumab are capable to cross the BBB with comparable effectiveness in the CNS, and their combination may be the most appropriate CNS metastasis treatment from EGFRm NSCLC. The CNS response rate in patients treated with osimertinib alone was 66% in the FLAURA study but 100% in the combined treatment with bevacizumab (12). In previous randomized clinical trials comparing osimertinib plus bevacizumab vs osimertinib alone, the combination arm failed to show prolongation of PFS in patients with advanced lung adenocarcinoma with EGFR mutation or EGFR T790M mutation. However, patients with symptomatic brain metastasis or LM were excluded from these clinical studies. As such, the therapeutic benefits of osimertinib plus bevacizumab for LM merit further investigation (18, 19).

Preclinical studies have confirmed that almonertinib has a higher intracranial concentration and good BBB penetration. Almonertinib has a longer survival time and lower toxicity in brain metastases treatment than osimitinib (6). At the 2021 ASCO annual meeting, the preliminary results of phase III AENEAS study demonstrated that almonertinib significantly improved PFS than gefitinib in the first-line treatment of patients with EGFRm advanced NSCLC (19.3 vs. 9.9 months, hazard ratios: 0.46) (20). Subgroup analysis showed that patients with brain metastases tended to benefit more from almonertinib treatment (hazard ratios = 0.38), which also verified the sufficient capacity of almonertinib to penetrate across BBB. Lu et al. (21) recently performed a phase II prospective study to evaluate the efficacy of osimertinib combined with bevacizumab for EGFRm NSCLC with LM and revealed that the median LM PFS and LM OS were 9.3 months and 12.6 months, respectively, which confirmed the significantly better survival advantage of combination therapy for LM than that of osimitinib monotherapy in the BLOOM study. However, no studies have investigated almonertinib combined with bevacizumab in patients with LM from EGFRm NSCLC. Here, almonertinib combined with bevacizumab firstly demonstrated favorable safety and effectiveness in EGFRm NSCLC with LM. However, further investigations with larger samples are needed to confirm this result due to the small case series.

## Data availability statement

The original contributions presented in the study are included in the article/supplementary material. Further inquiries can be directed to the corresponding author.

## Ethics statement

The studies involving human participants were reviewed and approved by the research ethics committee of the Nanjing Chest Hospital (Nanjing, China). The patients/participants provided their written informed consent to participate in this study. Written informed consent was obtained from the individual(s) for the publication of any potentially identifiable images or data included in this article.

## Author contributions

All authors listed have made a substantial, direct, and intellectual contribution to the work and approved it for publication.

## Acknowledgments

The authors thank the patients for their participation in the study and agreeing to the publishing of the report.

## Conflict of interest

The authors declare that the research was conducted in the absence of any commercial or financial relationships that could be construed as a potential conflict of interest.

The reviewer TL declared a shared parent affiliation with the authors to the handling editor at the time of review.

## Publisher’s note

All claims expressed in this article are solely those of the authors and do not necessarily represent those of their affiliated organizations, or those of the publisher, the editors and the reviewers. Any product that may be evaluated in this article, or claim that may be made by its manufacturer, is not guaranteed or endorsed by the publisher.

## Abbreviations

CNS, Central nervous system; CR, Complete response; CSF, Cerebrospinal fluid; CT, Computed tomography; LM, Leptomeningeal metastasis; MRI, Magnetic resonance imaging; OS, Overall survival; PFS, Progression-free survival; PS, Performance status; TKI, Tyrosine kinase inhibitors; VEGF, Vascular endothelial growth factor.
